# c-Jun Amino Terminal Kinase Signaling Promotes Aristolochic Acid-Induced Acute Kidney Injury

**DOI:** 10.3389/fphys.2021.599114

**Published:** 2021-02-12

**Authors:** Fan Yang, Elyce Ozols, Frank Y. Ma, Khai Gene Leong, Greg H. Tesch, Xiaoyun Jiang, David J. Nikolic-Paterson

**Affiliations:** ^1^Department of Nephrology, Monash Health and Monash University Centre for Inflammatory Diseases, Monash Medical Centre, Clayton, VIC, Australia; ^2^Department of Pediatrics, The First Affiliated Hospital of Sun Yat-sen University, Guangzhou, China

**Keywords:** acute kidney injury, chronic kidney disease, inflammation, c-Jun amino terminal kinase, macrophage, renal fibrosis, senescence

## Abstract

Aristolochic acid (AA) is a toxin that induces DNA damage in tubular epithelial cells of the kidney and is the cause of Balkan Nephropathy and Chinese Herb Nephropathy. In cultured tubular epithelial cells, AA induces a pro-fibrotic response *via* the c-Jun amino terminal kinase (JNK) signaling pathway. This study investigated the *in vivo* role of JNK signaling with a JNK inhibitor (CC-930) in mouse models of acute high dose AA-induced kidney injury (day 3) and renal fibrosis induced by chronic low dose AA exposure (day 22). CC-930 treatment inhibited JNK signaling and protected from acute AA-induced renal function impairment and severe tubular cell damage on day 3, with reduced macrophage infiltration and expression of pro-inflammatory molecules. In the chronic model, CC-930 treatment inhibited JNK signaling but did not affect AA-induced renal function impairment, tubular cell damage including the DNA damage response and induction of senescence, or renal fibrosis; despite a reduction in the macrophage pro-inflammatory response. In conclusion, JNK signaling contributes to acute high dose AA-induced tubular cell damage, presumably *via* an oxidative stress-dependent mechanism, but is not involved in tubular atrophy and senescence that promote chronic kidney disease caused by ongoing DNA damage in chronic low dose AA exposure.

## Introduction

Chinese herbal medicine is widely used for the prevention, treatment, and cure of a wide range of diseases (Yang et al., 2018). However, some traditional herbal medicines are toxic to the kidney such as aristolochic acids (AAs). Kidney damage associated with herbal medicines includes acute kidney injury, chronic kidney disease, nephrolithiasis, and bladder cancer (Yang et al., 2018). AA was identified as the nephrotoxin responsible for Balkan Nephropathy where the toxin was derived from weeds growing in fields that contaminated flour used in baking (Jadot et al., 2017). Indeed, the wide-spread recognition of kidney damage caused by this nephrotoxin has led to the term, aristolochic acid nephropathy (AAN).

Aristolochic acid is taken up by tubular epithelial cells of the kidney *via* the organic anion transporter OAT1/3 (Bakhiya et al., 2009). Toxicity to tubular epithelial cells is the hallmark of AAN which features atrophy and loss of tubules with extensive interstitial fibrosis with an inflammatory infiltrate, although glomeruli are largely spared (Vanherweghem et al., 1993). Analysis of 300 cases of Chinese Herb Nephropathy identified both acute kidney injury and more slowly progressive chronic kidney disease, which were associated with high or low levels of AA ingestion, respectively (Yang et al., 2012). Consistent with clinical findings, animal studies have shown that administration of a single high dose of AA can induce acute kidney injury with tubular necrosis, while repeated administration of low doses of AA leads to chronic kidney disease with tubular atrophy and interstitial fibrosis (Zhou et al., 2010a,b).

Upon uptake into cells, AA binds to DNA causes AA-DNA adducts which can result in a A:T→T:A transversion, inducing the DNA damage response and leading to cancer development over time (Jadot et al., 2017). In addition, studies using cultured tubular epithelial cells have shown that treatment with AA induces high levels of reactive oxygen species (ROS), and that AA-induced cell death can be suppressed using anti-oxidant approaches (Yu et al., 2011; Jadot et al., 2017). Animal studies support a mechanism of AA-induced ROS in tubular cell damage (Li et al., 2012), emphasizing the importance of targeting the response to oxidative stress in preventing the nephrotoxic effects of AA.

The c-Jun amino terminal kinase (JNK) enzyme is exquisitely sensitive to ROS and is known as a stress-activated protein kinase (Grynberg et al., 2017). Treatment of cultured tubular epithelial cells with AA leads to activation of the JNK enzyme, and the addition of a JNK inhibitor compound can inhibit AA-induced production of TGF-β1, upregulation of α-SMA, increased collagen I expression and cell cycle arrest (Yang et al., 2010; Zhou et al., 2010a; Rui et al., 2012). One study has used Western blotting to identify increased phosphorylation of the JNK enzyme in a model of AAN (Chang et al., 2020); however, the location of JNK activation and the functional significance of JNK activation in AAN remain unknown. Therefore, the aim of this study was to determine the pathological role of JNK signaling in both acute and chronic forms of AA-induced renal injury.

## Materials and Methods

### Animals

Male C57BL/6J mice of 8–12 weeks of age were obtained from the Monash Animal Research Platform (Clayton, VIC, Australia). Animal studies were approved (MMCB/2017/21) by the Monash Medical Centre Animal Ethics Committee and performed according to the Australian Code of Practice for the Care and Use of Animals for Scientific Purposes.

### Aristolochic Acid-Induced Acute Kidney Injury

Groups of seven or eight male mice were given a single intraperitoneal injection of 5 mg/kg aristolochic acid (Sigma-Aldrich, Castle Hill, NSW, Australia) dissolved in saline and killed 3 days later. Groups of AA-injected mice were: (i) treated with 75 mg/kg CC-930 (supplied by Celgene Corporation, San Diego, CA, United States) by twice daily oral gavage starting 1 h before AA injection and continuing until being killed on day 3 (termed CC-930 AA), (ii) treated with vehicle only (0.5% carboxymethylcellulose in water) by twice daily oral gavage (termed vehicle AA), or (iii) did not receive any treatment (termed AA or untreated AA). The 75 mg/kg BID dosing of CC-930 was based on a previous study (Reich et al., 2012). In addition, one group of normal mice (no experimentation) was used as controls. Blood was collected at the time of killing. Urine samples were collected the day before killing. Serum and urine creatinine levels and urine total protein levels were measured using a Duppon ARL Analyzer at the Department of Clinical Biochemistry, Monash Health.

### Aristolochic Acid-Induced Chronic Kidney Injury

Groups of 9 or 10 male mice were given intraperitoneal injections of 2 mg/kg AA every second day from day 0 until being killed on day 22. Groups of AA-injected mice were treated with 75 mg/kg CC-930, or vehicle alone, by twice daily oral gavage starting 1 h before AA injection and continuing until being killed on day 22. Normal mice (no experimentation) were used as controls.

### Histology

Slices of kidney tissue were fixed in 4% neutral-buffered formalin or methylcarn fixative and processed for embedding in paraffin. Periodic acid-Schiff (PAS) plus hematoxylin staining was performed on 2 μm sections of formalin-fixed kidney tissue. Acute tubular injury was scored in the day 3 AA model as follows. Tubular cross-sections across the entire cortex were analyzed under high power (×400) and scored as normal or damaged. The definition of tubular damage included one or more of the following: tubular dilation or atrophy, loss of brush border, loss of tubular nuclei, and cast formation. Scoring was performed on blinded slides.

### Immunohistochemistry

Immunostaining with the F4/80 antibody to detect macrophages (Bio-Rad, Gladesville, NSW, Australia), with a goat anti-collagen IV antibody (Southern Biotechnology, Birmingham, AL, United States) and with rabbit anti-α-SMA (Abcam, Melbourne, VIC, Australia) was carried out on 4 μm sections of methylcarn-fixed tissue using an avidin-biotin complex system with horseradish peroxidase and the substrate diaminobenzidine. Immunostaining with rabbit antibodies to phospho-c-Jun Ser63, phospho-Histone 2A.X Ser139, and CDKN1A (all from Cell Signaling, San Diego, CA, United States) was carried out on 4 μm sections of formalin-fixed sections after antigen retrieval (some sections had a PAS counterstain) as previously described (Flanc et al., 2007). The area of collagen IV staining was assessed by point counting of the entire cortex under medium power (x200) with a minimum of 1,200 points evaluated. Scoring was performed on blinded slides.

### Western Blotting

Frozen kidney samples were homogenized in 0.5 ml lysis buffer and processed for Western blotting as previously described (Ma et al., 2014). Blots were probed for phospho-c-Jun and α-tubulin as the loading control using goat anti-rabbit Alexa Fluor 680 or donkey anti-mouse IRDye 800 secondary antibodies (Molecular Probes) and detected using the Odyssey Infrared Image Detecting System (LICOR). Densitometry analysis used ImageJ software, NIH.

### Real Time Polymerase Chain Reaction

RNA extraction from frozen kidney tissue, cDNA synthesis, and PCR reactions on a StepOne Real-Time PCR System (Applied Biosystems, Foster City, CA, United States) were performed as previously described (Hou et al., 2018). Taqman primer/probes for NOS2 and α-SMA have been described previously (Ma et al., 2011), and other primer/probes were purchased from Applied Biosystems. The comparative Ct (*Δ*Ct) method was used to quantify the relative amount of mRNA which was normalized against the internal *Gapdh* mRNA control (Applied Biosystems).

### Statistical Analysis

Data are shown as mean ± SD. Analysis used one-way ANOVA with Tukey’s multiple comparison test, except for analysis of groups of two which used the student’s *t*-test. Analysis was performed using GraphPad Prism 8.0 (San Diego, CA, United States).

## Results

### Acute Kidney Injury on Day 3 After AA Administration

**Figure 1:**
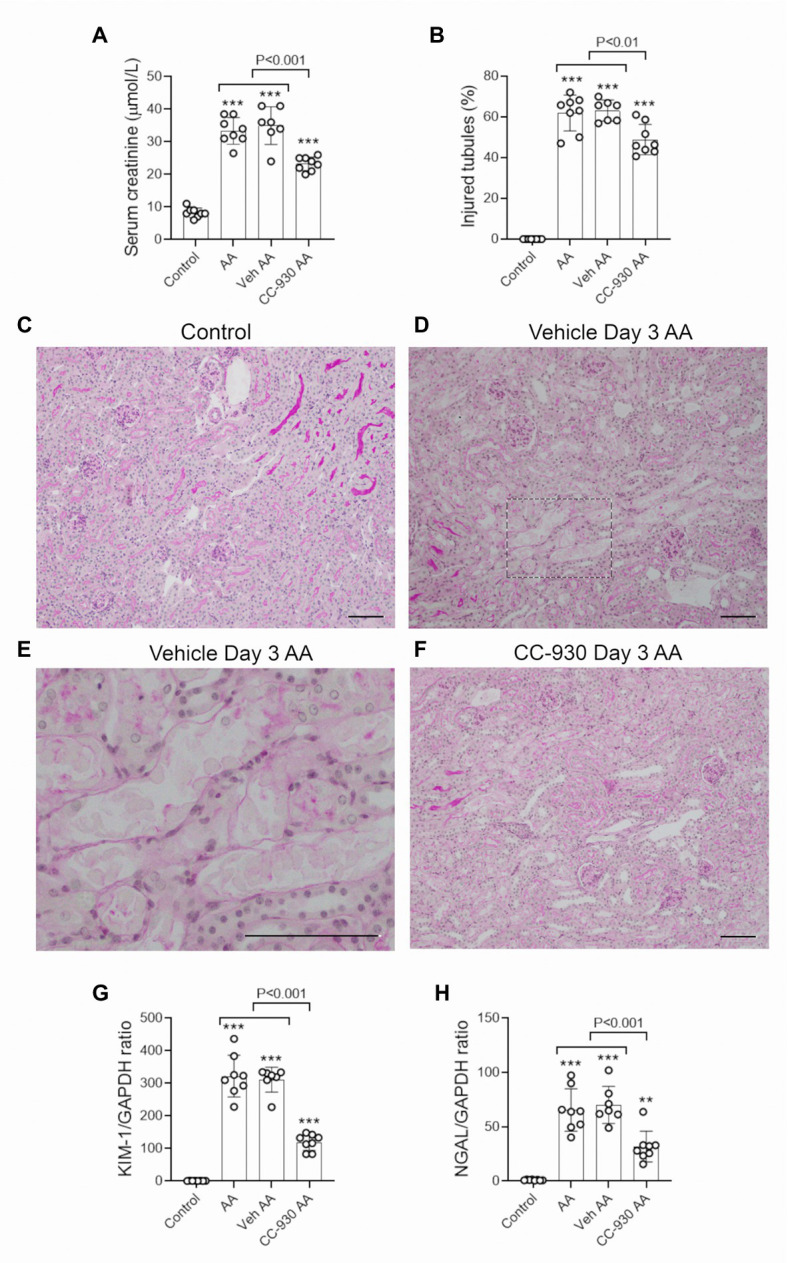
Renal function and tubular damage on day 3 of aristolochic acid (AA)-induced acute kidney injury. AA-injected mice were either untreated (AA), or treated with vehicle (Veh AA) or CC-930 (CC-930 AA) and compared to normal controls. **(A)** Serum creatinine levels. **(B)** Graph of tubular damage score. (**C**–**F**) Periodic acid-Schiff (PAS) staining of kidney sections. **(C)** Kidney structure in normal control. **(D)** Vehicle treated AA showing damaged tubules with loss of brush border, loss of tubular nuclei and sloughing of cells into the lumen. **(E)** High power view of the image in D. **(F)** CC-930 treated AA shows less severe tubular damage compared to vehicle treated. Bars represent 100 μm. Reverse transcription PCR (RT-PCR) analysis of mRNA levels for the tubular damage markers; **(G)** KIM-1/HAVCR1 and **(H)** NGAL/LCN2. One-way ANOVA with Tukey’s multiple comparison test. ^**^p < 0.01 and ^***^p < 0.001 vs. control.

A single injection of 5 mg/kg AA resulted in an acute loss of kidney function, as shown by a 4-fold increase in serum creatinine levels (Figure 1A). Compared to normal (control) kidney, PAS staining showed significant tubular damage in vehicle and untreated groups on day 3. Tubular damage featured loss of the brush border, loss of tubular nuclei and sloughing of cells into the tubular lumen (Figures 1B–E). Cellular damage was most obvious in the proximal portion of the tubule. There was also a marked increase in the mRNA levels of the tubular damage markers, Kidney Injury Molecule 1 (KIM1/HAVCR1) and Lipocalin 2 (LCN2/NGAL) in untreated and vehicle treated AA groups on day 3 (Figures 1G,H).

**Figure 2:**
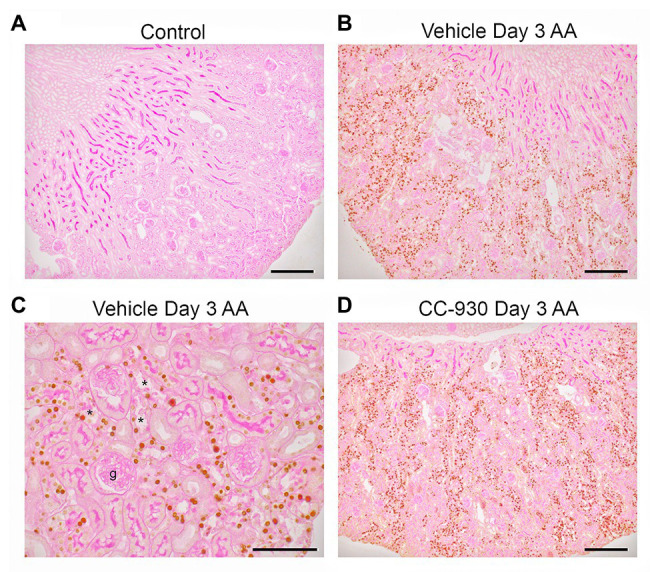
Immunostaining for the DNA damage marker, phospho-Histone 2A.X Ser^139^, on day 3 of AA-induced acute kidney injury. **(A)** No staining is seen the normal control kidney. **(B)** Many tubular nuclei show p-H2A.X staining in the cortex in vehicle treated day 3 AA. **(C)** High power view of vehicle treated day 3 AA shows staining in both damaged (^*^) and intact tubules, but no staining is seen in the glomerular tuft (g). **(D)** A similar pattern of stained for p-H2A.X in tubular nuclei is seen in CC-930 treated day 3 AA. Sections have PAS counterstain. Bars represent 200 μm **(A,B,D)**, or 100 μm **(C)**.

**Figure 3:**
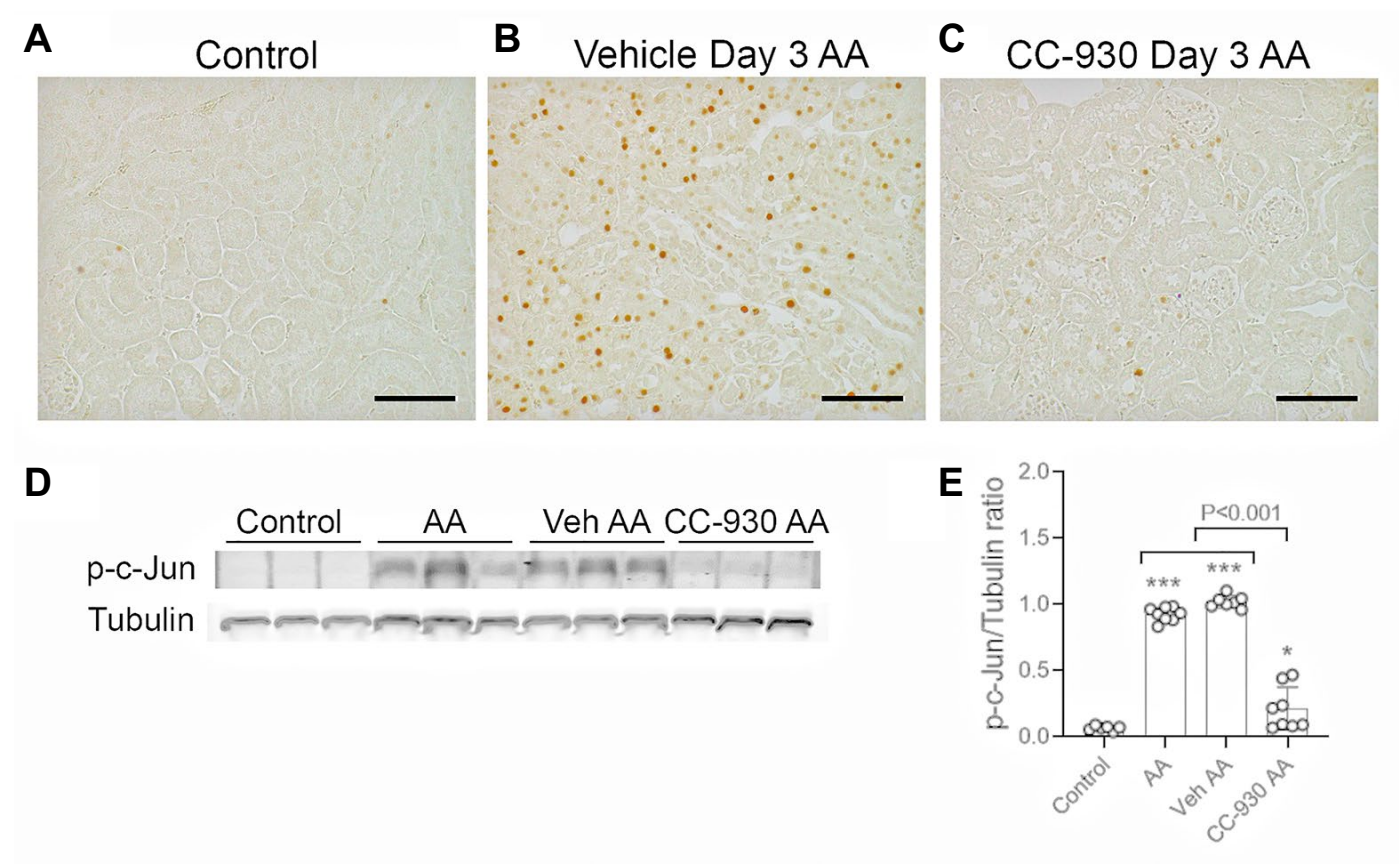
c-Jun amino terminal kinase (JNK) signaling on day 3 of aristolochic acid (AA) induced acute kidney injury. AA injected mice were either untreated (AA), or treated with vehicle (Veh AA) or CC-930 (CC-930 AA) and compared to normal controls. **(A-C)** Immunohistochemistry staining for phospho-c-Jun Ser^63^. **(A)** Normal control kidney shows only very occasion p-c-Jun+ cells. **(B)** Vehicle treated AA shows large numbers of p-c-Jun+ nuclei, predominantly in tubular epithelial cells, including in damaged tubules. **(C)** CC-930 treated AA showing a dramatic reduction in p-c-Jun+ cells. Bars represent 100 μm. **(D)** Western blot of kidney lysates for p-c-Jun and reprobed for α-tubulin. **(E)** Graph showing quantification of Western blots. One-way ANOVA with Tukey’s multiple comparison test. ^*^p < 0.05 and ^***^p < 0.001 vs. control.

Aristolochic acid is known to cause DNA damage by inducing adducts (Jadot et al., 2017). DNA damage rapidly induces phosphorylation of the H2A.X variant which is required for checkpoint-mediated cell cycle arrest and DNA repair (Yuan et al., 2010). While no phospho-H2A.X Ser^139^ staining was evident in control kidney, many tubular cells showed strong nuclear staining for p-H2A.X in vehicle treated and untreated day 3 AA. High power shows p-H2A.X staining in remaining nuclei in damaged tubules, although tubules without obvious damage also showed staining. Of note, very few cells in glomeruli or in the early proximal tubule were stained (Figures 2A–C).

c-Jun amino terminal kinase is the only enzyme that phosphorylates c-Jun at Serine 63, allowing detection of p-c-Jun Ser^63^ as a surrogate marker of JNK activation (Pulverer et al., 1991; Minden et al., 1994; Ma et al., 2007). Only very occasional cells were stained for p-c-Jun in normal mouse kidney. By contrast, many p-c-Jun+ cells are seen in the kidney of vehicle and untreated groups on day 3 after AA (Figures 3A,B). The p-c-Jun staining is prominent in all parts of the nephron, including damaged tubules and tubules without obvious histologic damage. Some interstitial p-c-Jun+ cells are also evident, whereas few p-c-Jun+ cells were evident in glomeruli. Western blotting shows a strong band for p-c-Jun in vehicle and untreated day 3 AA groups, which was barely detected in normal kidney (Figures 3D,E).

**Figure 4:**
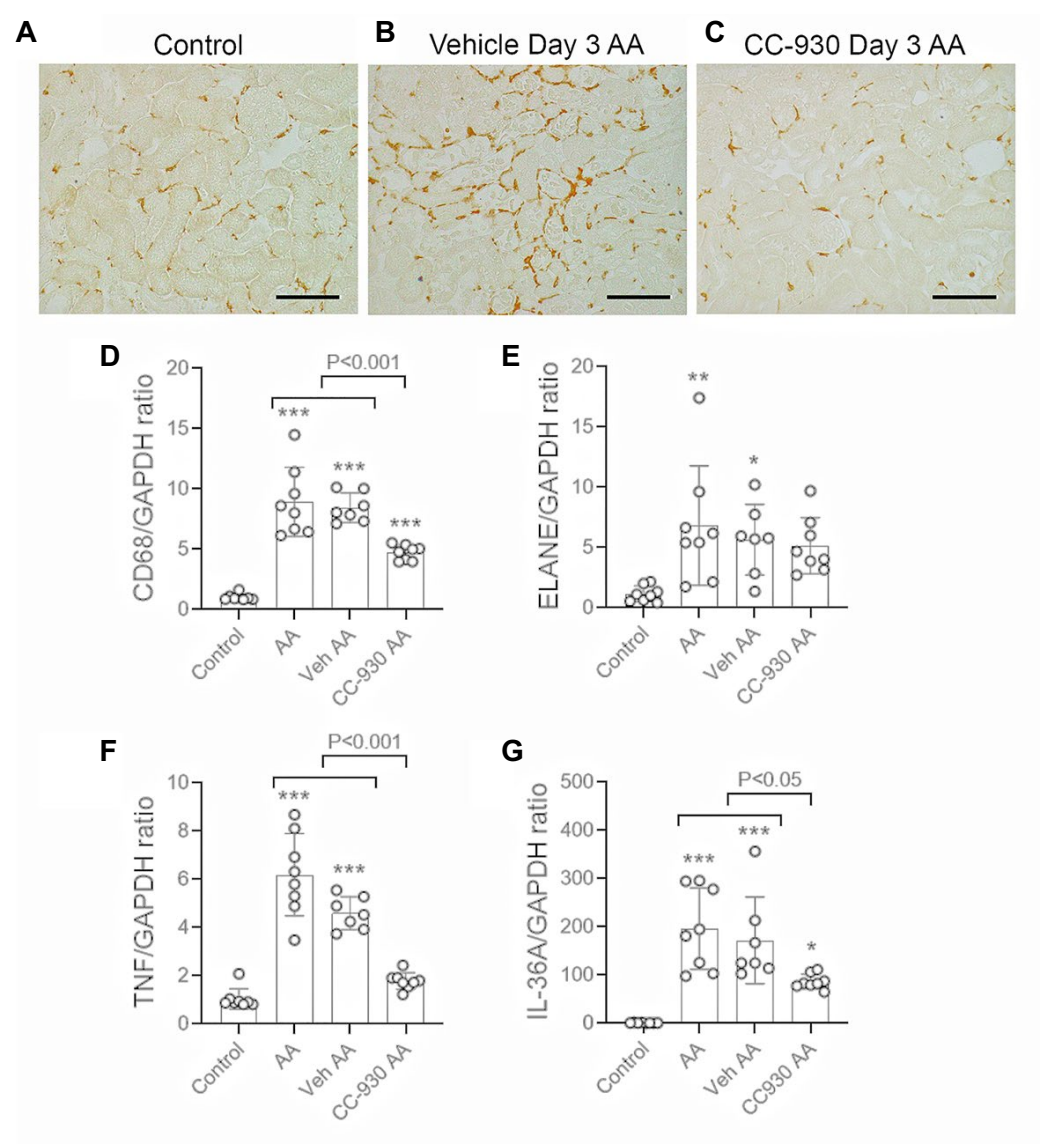
Macrophage infiltration and inflammation on day 3 of AA-induced acute kidney injury. AA injected mice were either untreated (AA), or treated with vehicle (Veh AA) or CC-930 (CC-930 AA) and compared to normal controls. **(A-C)** Immunohistochemistry staining for F4/80+ macrophages. **(A)** Normal control kidney shows a regular network of resident kidney macrophages. **(B)** Vehicle treated AA shows a significant increase in F4/80+ macrophages, mainly around damaged tubules. **(C)** CC-930 treated AA showing a reduction in the number of infiltrating macrophages. Bars represent 100 μm. (**D**–**G**) RT-PCR analysis of kidney tissue for: **(D)** CD68; **(E)** ELANE; **(F)** TNF, and **(G)** IL-36A. One-way ANOVA with Tukey’s multiple comparison test. ^*^p < 0.05, ^**^p < 0.01, and ^***^p < 0.001 vs. control.

Inflammation was evident on day 3 after AA administration in vehicle and untreated groups. Immunostaining showed a focal infiltrate of F4/80+ macrophages in areas of tubular damage (Figures 4A,B). This was consistent with the 8-fold increase in mRNA levels of the macrophage marker, CD68 (Figure 4D). There was a significant, but variable, neutrophil infiltrate as shown by mRNA levels for neutrophil elastase/ELANE (Figure 4E). Leukocyte infiltration was associated with up-regulation of inflammatory cytokines TNF and IL-36*α* (Figures 4F,G).

### Effect of CC-930 Treatment on Acute Kidney Injury on Day 3 in the AA Model

CC-930 treatment gave protection from acute kidney injury as shown by a significant reduction in serum creatinine levels compared to the untreated and vehicle treated groups (Figure 1A). CC-930 treatment also significantly reduced tubular damage as shown by scoring of PAS stained sections and by the mRNA levels of KIM-1 and NGAL (Figures 1B,F–H). As expected, CC-930 treatment did not affect AA-induced DNA damage, as shown by a lack of effect upon p-H2A.X staining (Figure 2D). However, this protection against acute kidney injury was associated with a profound reduction in JNK signaling as demonstrated by reductions in c-Jun phosphorylation using both immunohistochemistry and Western blotting (Figures 3C–E).

CC-930 treatment significantly reduced the macrophage infiltrate based on F4/80 immunostaining and CD68 mRNA levels (Figures 4C,D), and reduced mRNA levels of TNF and IL-36α (Figures 4F,G). However, the CC-930 did have a significant effect upon the variable neutrophil infiltrate (Figure 4E).

### Characterization of Chronic Kidney Disease Induced by Repeated Administration of AA

**Figure 5:**
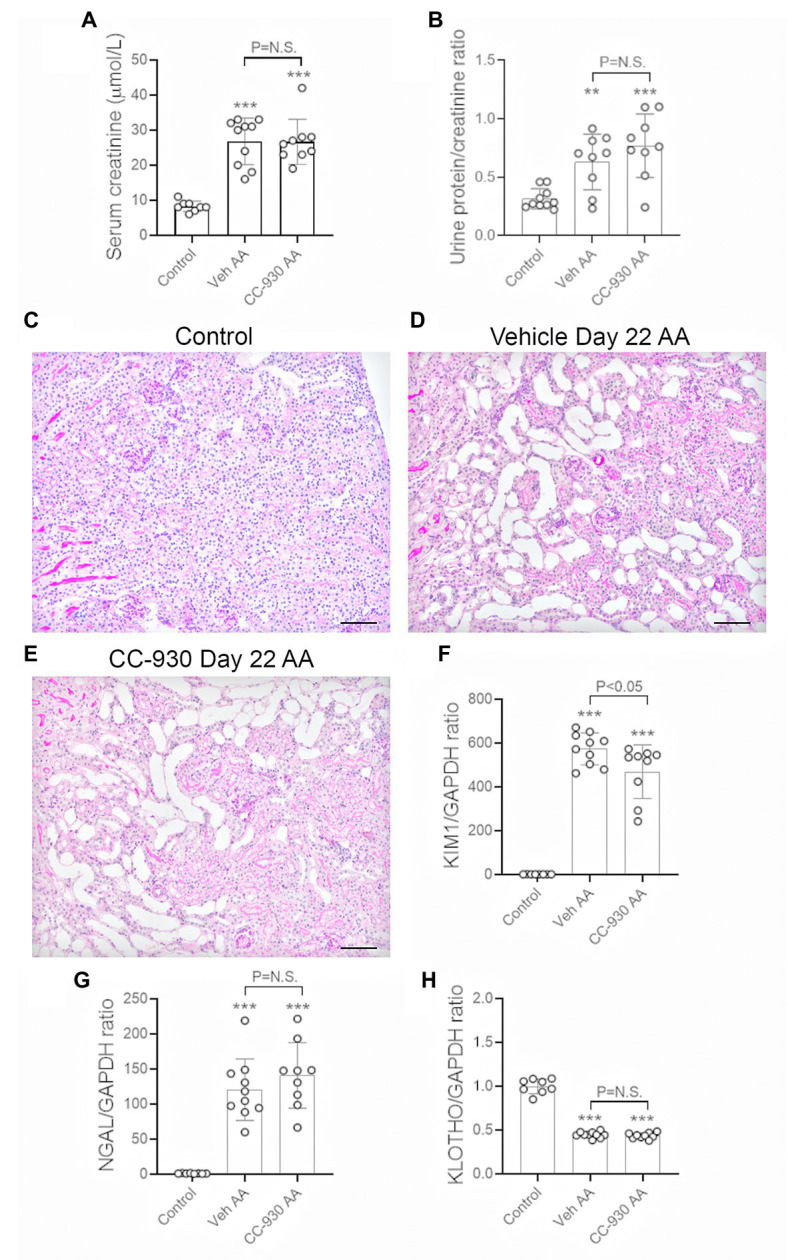
Renal function and tubular damage on day 22 of aristolochic acid (AA) induced chronic kidney disease. Vehicle and CC-930 treated AA groups were compared to normal controls. **(A)** Serum creatinine levels. **(B)** Urinary protein excretion measured as a ratio of mg total protein/mol creatinine. **(C)** PAS staining of normal control kidney. **(D)** PAS staining of vehicle treated AA shows marked tubular atrophy, interstitial cell infiltration and occasional tubular casts, while glomeruli are largely normal. **(E)** PAS staining of CC-930 treated AA shows similar chronic tubular damage to that in vehicle treated. Bars represent 100 μm. RT-PCR analysis of: **(F)** KIM-1/HAVCR1, **(G)**, NGAL/LCN2, and **(H)** α-Klotho mRNA levels. One-way ANOVA with Tukey’s multiple comparison test. ^**^p < 0.01 and ^***^p < 0.001 vs. control. N.S., not significant.

A model of chronic tubulointerstitial disease was induced by repeated administration of a lower dose of AA (2 mg/kg) every second day from day 0 until being killed on day 22. This resulted in impaired kidney function in vehicle treated mice as shown by a 3-fold increase in serum creatinine over that of normal mice (Figure 5A), and a modest increase in urinary protein excretion (Figure 5B). Histologic analysis showed marked tubular atrophy, accumulation of cells in the interstitial space, and some tubular casts. By contrast, glomeruli remained largely normal (Figures 5C,D). Substantial tubular damage was also indicated by a marked increase in mRNA levels of tubular damage markers KIM-1 and NGAL, while expression of the tubular protective molecule, α-Klotho, was substantially reduced (Figures 5F–H).

**Figure 6:**
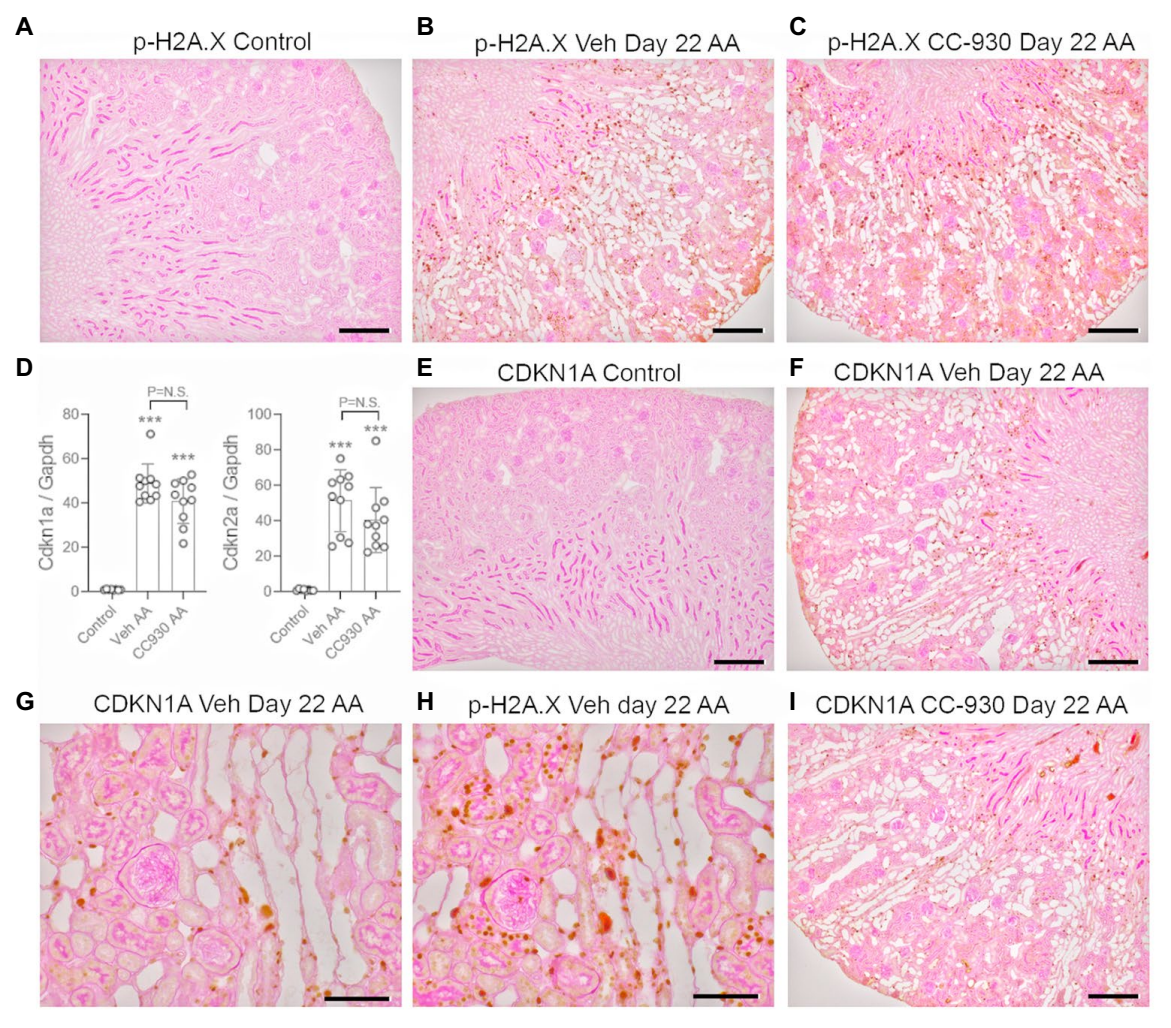
DNA damage (phospho-H2A.X) and senescence markers (CDKN1A) on day 22 of aristolochic acid (AA) induced chronic kidney disease. **(A)** No staining for p-H2A.X is seen the normal control kidney. **(B)** Numerous p-H2A.X stained nuclei are seen in vehicle treated day 22 AA, evident in both atrophied and normal tubules. **(C)** A similar pattern of p-H2A.X nuclear staining is seen in CC-930 treated day 22 AA. **(D)** RT-PCR analysis of kidney tissue for Cdkn1a and Cdkn2a. **(E)** No staining for CDKN1A is seen the normal control kidney. **(F)** Nuclear staining for CDKN1A is evident in cells within atrophied tubules in vehicle treated day 22 AA. **(G)** Higher power view emphasizes that CDKN1A staining is mostly restricted to damaged tubules. **(H)** Serial section to **(F)** showing p-H2A.X staining is also evident damaged tubules, but is more extensive than that of CDKN1A. **(I)** CDKN1a staining in CC-930 treated day 22 AA is similar to that seen in vehicle treated day 22 AA. Bars represent 200 μm (**A**–**C,E,F,I**), or 100 μm **(G,H)**. One-way ANOVA with Tukey’s multiple comparison test. ^***^p < 0.001 vs. control; N.S., not significant.

Chronic administration of low dose AA caused sustained activation of the DNA damage response as shown by immunostaining for p-H2A.X (Figures 6A,B), although fewer cells were stained compared to the acute effects of high dose AA on day 3 (Figure 2B). Only very low mRNA levels for the senescence markers, Cyclin Dependent Kinase Inhibitor 1A (Cdkn1a/p21^CIP1^) and Cdkn2a/p16^INK4A^ were evident in control kidney, but both markers were highly upregulated in vehicle treated day 22 AA (Figure 6D). No cells were stained for CDKN1A in control kidney, but cell nuclei in atrophic tubules were stained in vehicle treated day 22 AA (Figures 6E,F). Analysis of serial sections show CDKN1A stained cells in the same damaged tubules that exhibit p-H2A.X stained cells, although overall more cells were stained for p-H2A.X than for CDKN1A (Figures 6G,H).

**Figure 7:**
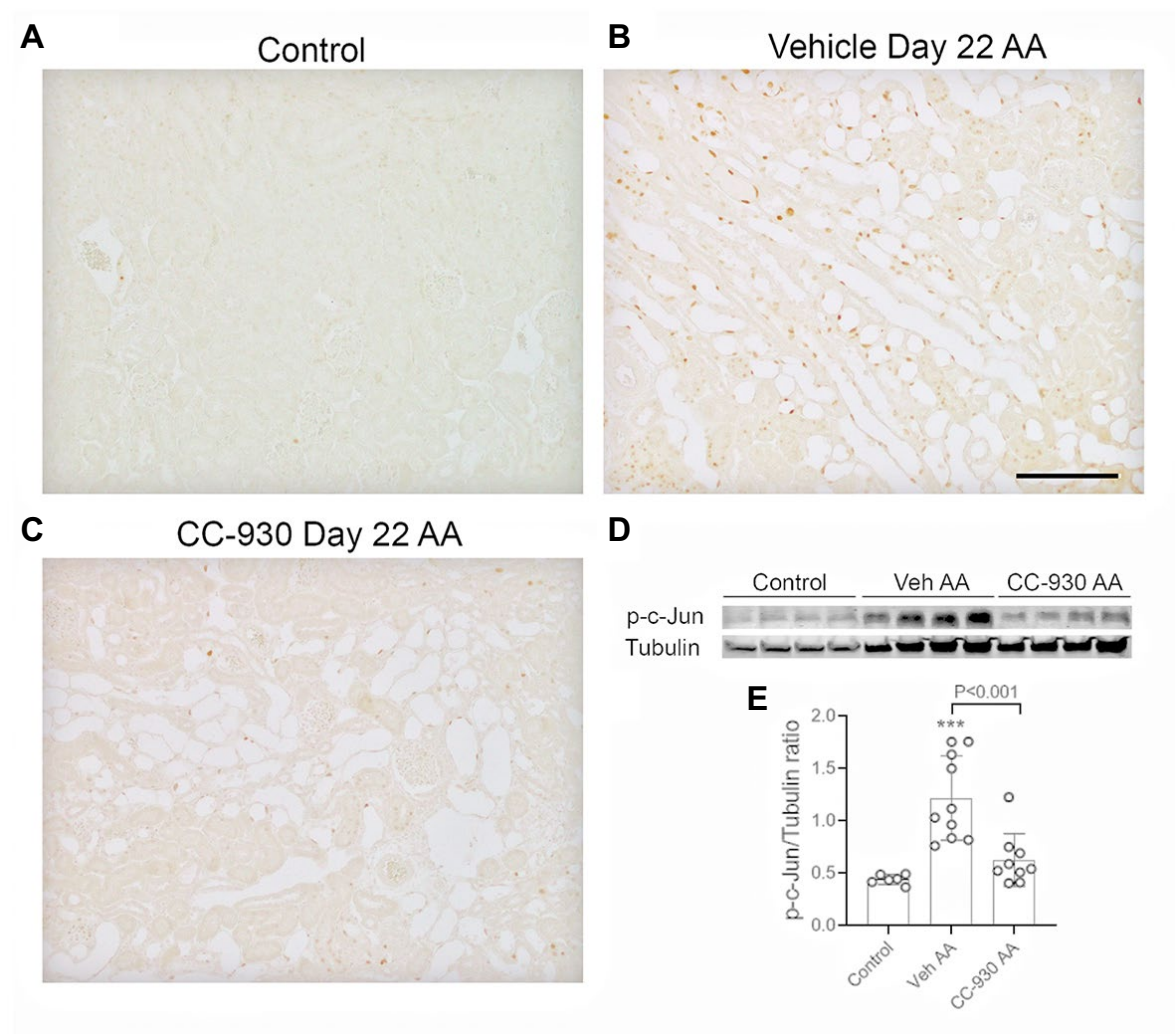
JNK signaling on day 22 of aristolochic acid (AA) induced chronic kidney disease. Vehicle and CC-930 treated AA groups were compared to normal controls. **(A–C)** Immunohistochemistry staining for phospho-c-Jun Ser^63^. **(A)** Normal control kidney shows very little p-c-Jun staining. **(B)** Vehicle treated AA shows tubular cells with nuclear staining for p-c-Jun, being most prominent in dilated and atrophic tubules. **(C)** CC-930 treated AA showing a very substantial reduction in p-c-Jun+ cells. Bar represent 100 μm. **(D)** Western blot of kidney lysates for p-c-Jun and reprobed for α-tubulin. **(E)** Graph showing quantification of Western blots. One-way ANOVA with Tukey’s multiple comparison test. ^***^p < 0.001 vs. control.

Chronic administration of the low dose AA also caused sustained activation of the JNK signaling pathway at day 22 in vehicle treated mice. Many p-c-Jun stained cells were evident, particularly in dilated and atrophic tubules, as well as some interstitial p-c-Jun+ cells (Figures 7A,B). In addition, a strong band for p-c-Jun was evident Western blots of vehicle treated mice on day 22 of chronic AA administration (Figures 7D,E).

**Figure 8:**
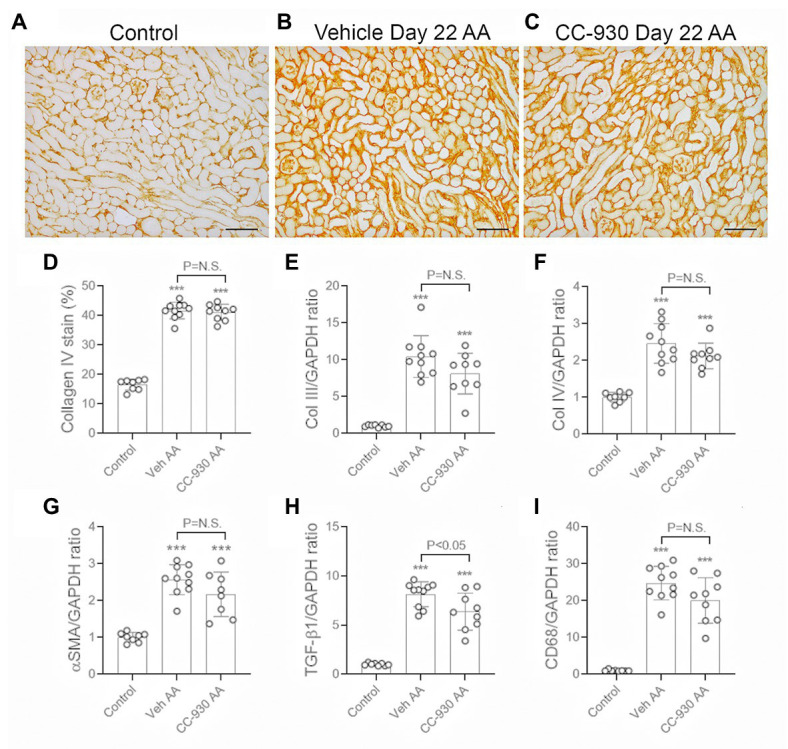
Renal fibrosis on day 22 of AA-induced chronic kidney disease. Vehicle and CC-930 treated AA groups were compared to normal controls. **(A–C)** Immunohistochemistry staining for collagen IV. **(A)** Normal mouse kidney showing collagen IV in the tubular and glomerular basement membranes. **(B)** Vehicle treated day 22 AA shows increased interstitial collagen IV staining, particularly in areas with atrophic tubules. **(C)** CC-930 treated day 22 AA shows a similar increase in interstitial collagen IV deposition as seen with vehicle treatment. Bars represent 100 μm. **(D)** Graph showing quantification of the area of interstitial collagen IV staining. RT-PCR analysis of: **(E)** Collagen III; **(F)** Collagen IV; **(G)** α-SMA/Acta2; **(H)** TGF-β1, and **(I)** CD68. One-way ANOVA with Tukey’s multiple comparison test. ^***^p < 0.001 vs. control. N.S., not significant.

**Figure 9:**
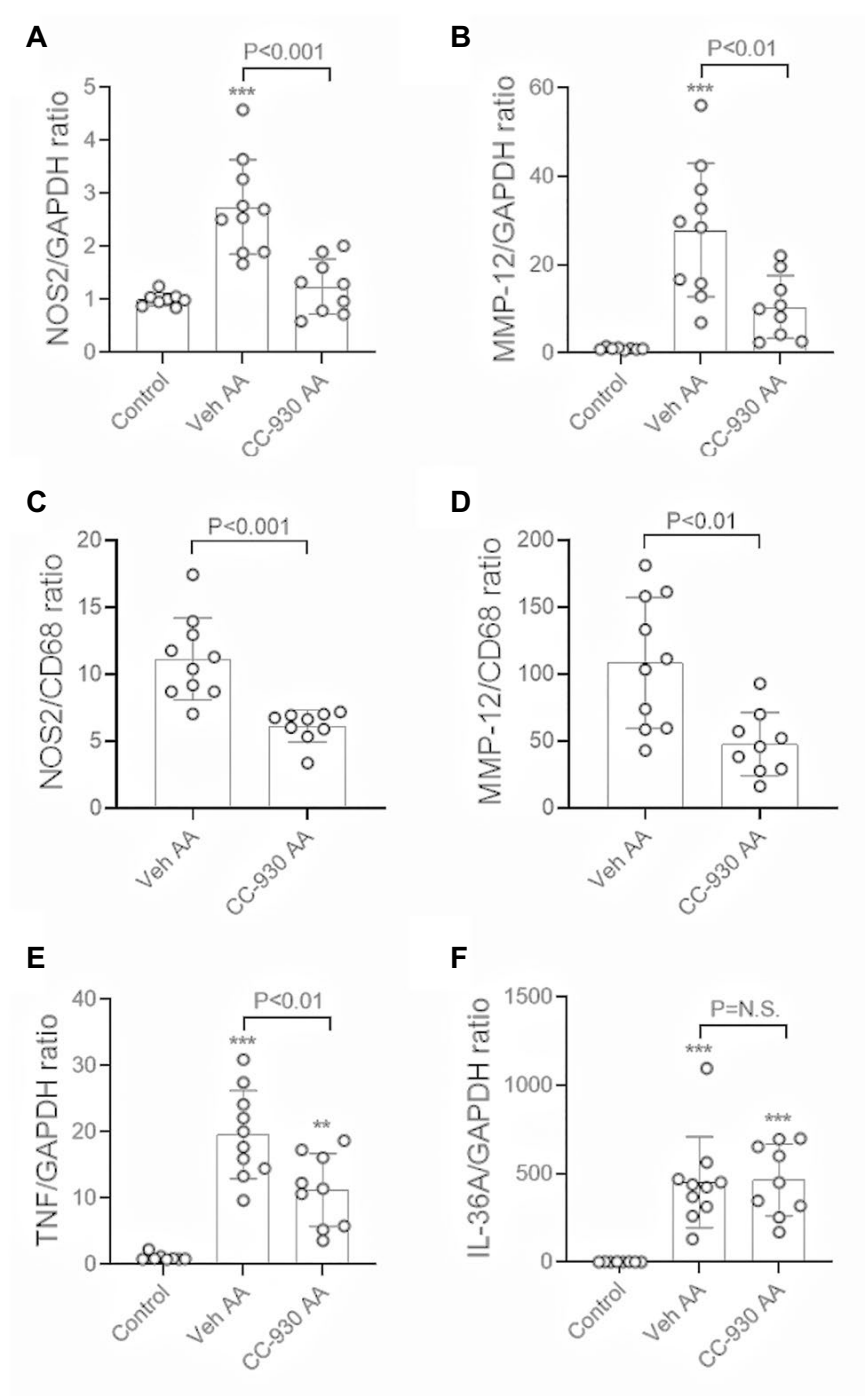
Macrophage activation and inflammation on day 22 of aristolochic acid (AA) induced chronic kidney disease. Vehicle and CC-930 treated AA groups were compared to normal controls. RT-PCR analysis of: **(A)** NOS2, and; **(B)** MMP-12. Graphs show the relative mRNA ratio of: **(C)** NOS2/CD68, and; **(D)** MMP-12/CD68. RT-PCR analysis of: **(E)** TNF, and; **(F)** IL-36A. One-way ANOVA with Tukey’s multiple comparison test, or student’s *t*-test. ^**^p < 0.001 and ^***^p < 0.001 vs. control. N.S., not significant.

Significant interstitial fibrosis was evident on day 22 of chronic AA administration. Increased collagen IV deposition was seen in areas of atrophic tubules (Figures 8A,B), which was associated with focal accumulation of α-SMA+ myofibroblasts (not shown). Image analysis showed a 2.5-fold increase in the interstitial deposition of collagen IV in the cortex in the vehicle treated day 22 AA group compared to normal controls (Figure 8D). This was accompanied by a significant increase in mRNA levels of collagen III and IV, α-SMA/Acta2, and TGF-β1 (Figures 8E–H). There was also a marked macrophage infiltrate as shown by the increase in CD68 mRNA levels (Figure 8I), with increases in mRNA levels of macrophage M1-type pro-inflammatory molecules NOS2 and MMP-12 (Figures 9A,B). In addition, vehicle treated mice on day 22 of AA administration showed increased expression of TNF and IL-36α (Figures 9E,F).

### Effect of CC-930 Treatment on AA-Induced Chronic Kidney Disease

CC-930 treatment of mice with chronic administration of AA did not affect the loss of kidney function as shown by serum creatinine levels or the development of mild proteinuria (Figures 5A,B). Consistent with this, CC-930 treatment did not affect histologic tubular damage or changes in NGAL or α-Klotho mRNA levels (Figures 5E,G,H), although the high levels of KIM-1 mRNA were significantly reduced by 18% (Figure 5F).

CC-930 treatment did not affect the sustained DNA damage response as shown by immunostaining for p-H2A.X (Figure 6C), or the induction of cell senescence as shown by Cdkn1a and Cdkn2a mRNA levels and immunostaining for CDKN1A (Figures 6D,I). However, CC-930 treatment did substantially reduce c-Jun phosphorylation as shown by both immunohistochemistry and Western blotting (Figures 7C–E).

CC-930 treatment had no significant effect on renal fibrosis on day 22 of AAN in terms of collagen IV deposition and mRNA levels of collagen III and IV, and α-SMA/Acta2 (Figures 8C–G), although there was a small reduction in the mRNA levels of TGF-β1 (Figure 8H).

CC-930 treatment did not affect the substantial macrophage infiltration on day 22 as shown by CD68 mRNA levels (Figure 8I). However, markers of M1-type macrophages, NOS-2 and MMP-12, were substantially reduced by CC-930 treatment and the NOS2:CD68 and MMP-12:CD68 mRNA ratios were also significantly reduced compared to the vehicle treated group (Figures 9A–D). Finally, CC-930 treatment significantly reduced TNF, but not IL-36α, mRNA levels (Figures 9E,F).

## Discussion

This study has shown that inhibition of JNK signaling with CC-930 provided significant protection against high dose AA-induced acute kidney injury, but failed to protect against chronic kidney disease induced by repeated low dose AA administration.

We identified a strong induction of JNK signaling in tubular epithelial cells on day 3 after a single high dose of AA based on amino-terminal phosphorylation of c-Jun at Ser^63^, validating previous studies showing that AA induces JNK activation in cultured tubular cells (Yang et al., 2010; Zhou et al., 2010a; Rui et al., 2012). We also demonstrated that chronic administration of low dose AA causes sustained JNK activation in tubular epithelial cells, particularly in atrophic tubular cells. Treatment with CC-930 was highly effective in suppressing JNK signaling.

The protection against acute AA-induced tubular cell damage and renal function impairment is consistent with other studies in which JNK inhibition suppressed acute kidney injury induced by renal ischemia/reperfusion injury (IRI) or cisplatin (Francescato et al., 2007; Wang et al., 2007; Kanellis et al., 2010). The production of ROS is a prominent feature in all three of these models of acute kidney injury, and studies have shown that blockade of JNK signaling prevents ROS-induced death of tubular epithelial cells (Pat et al., 2003; Arany et al., 2004), providing direct evidence that JNK is critical in ROS-induced tubular cell death. While CC-930 treatment did not affect the AA-induced DNA damage response, the protective effect of CC-930 seen in the acute AA model is most likely due to inhibition of tubular damage and cell death caused by the oxidative stress induced by high dose AA. Indeed, this mechanistic link between DNA damage and ROS production is supported by the presence of cells exhibiting JNK signaling and the DNA damage response within damaged tubules.

CC-930 treatment also reduced leukocyte infiltration and the expression of inflammatory cytokines in AA-induced acute kidney injury – a finding consistent with the suppression of inflammation seen with JNK inhibition in the IRI and cisplatin models of acute kidney injury (Francescato et al., 2007; Wang et al., 2007; Kanellis et al., 2010). This may simply reflect the reduction in tubular damage and consequent reduced release of pro-inflammatory and chemotactic danger-associated molecular patterns. Alternatively, this could reflect a specific role for JNK signaling *via* Activator Protein 1 (AP1) in promoting kidney expression of inflammatory molecules since transcription of *Nos2*, *Tnf*, and *Mmp12* genes has been shown to operate *via* AP1 (Newell et al., 1994; Wu et al., 2001; Lin et al., 2007; Grynberg et al., 2017).

Given the significant protective effect of JNK inhibition in AA-induced acute kidney injury, we were surprised by the lack of protection seen with CC-930 in AA-induced chronic kidney disease. In particular, cell culture studies have shown that short-term exposure of tubular epithelial cells to AA induces a strong pro-fibrotic response with increased expression of TGF-β1 and α-SMA, and increased production of collagens (Yang et al., 2010; Zhou et al., 2010a; Rui et al., 2012). In addition, JNK blockade has been shown to reduce inflammation and fibrosis across different models of chronic kidney disease. For example, JNK blockade suppressed tubular cell death, inflammation and the aggressive fibrosis seen in unilateral ureteric obstruction (Ma et al., 2007). JNK blockade prevents the development of crescentic glomerulonephritis in susceptible rats with protection from renal impairment, glomerular damage, and inhibition of the macrophage pro-inflammatory response and renal fibrosis (Flanc et al., 2007). Indeed, intervention in established crescentic disease with a JNK inhibitor still provides significant protection against inflammation and renal fibrosis (Ma et al., 2009). Furthermore, in two other classic models of renal interstitial fibrosis – unilateral IRI and folic acid-induced fibrosis – administration of a JNK inhibitor can suppress both inflammation and fibrosis (de Borst et al., 2009; Jiang et al., 2019).

One possible explanation for the protective effects of JNK inhibition in acute but not chronic AA-induced renal injury is that a different underlying pathogenic mechanism drives chronic injury. Thus, while JNK signaling is important for high dose AA-induced ROS production and cell damage in acute kidney injury, this may have only a minor role in the response to the accumulation of DNA damage occurring in ongoing exposure to low dose AA-induced chronic kidney disease. AA is a potent inducer of the DNA damage response in cultured tubular epithelial cells (Chen et al., 2010a,b). We validated this finding *in vivo* with clear activation of the DNA damage response (phosphorylation of H2A.X) in tubular epithelial cells following acute exposure to high dose AA on day 3, and ongoing activation of the DNA damage response with chronic exposure to low dose AA on day 22. Indeed, DNA damage is potent inducer of cellular senescence (Docherty et al., 2019), and senescent tubular epithelial cells have been implicated in the progression of renal interstitial fibrosis (Kishi et al., 2019). We found the induction of numerous senescent cells in atrophied tubules on the basis of CDKN1A expression and marked upregulation of Cdkn1a and Cdkn2a mRNA levels on day 22 of chronic AA administration. Indeed, the same atrophied tubules contained epithelial cells with activation of the DNA damage response and expression of senescence markers. Many cells in these atrophic tubules also exhibited JNK signaling, but JNK inhibition did not affect either the DNA damage response or the induction of senescence in these damage tubules – providing a rationale for the failure of JNK blockade to suppress renal fibrosis in this setting.

A second possible mechanism driving chronic AA-induced kidney injury is TGF-β/Smad3 signaling (Zhou et al., 2010a). While JNK has been shown to phosphorylate Smad3 to enhance the fibrotic response in short term AA stimulation of tubular epithelial cells (Zhou et al., 2010a), and combined JNK and Smad3 inhibition gives additive benefit in reducing folic acid-induced renal fibrosis (Jiang et al., 2019); it may that chronic administration of AA drives a Smad3-dependent fibrosis that is largely independent of JNK signaling.

Our previous studies found that global macrophage depletion using a c-fms inhibitor suppressed chronic kidney disease in response to repeated AA administration (Dai et al., 2016). This is consistent with other models of kidney disease in which macrophages play an important role in both tubular damage and renal interstitial fibrosis (Han et al., 2013; Meng et al., 2016; Tang et al., 2019). JNK signaling in macrophages has been shown to induce a pro-inflammatory M1 response that induces glomerular and tubulointerstitial renal injury in experimental glomerulonephritis (Ikezumi et al., 2004; Flanc et al., 2007). In the current study, JNK inhibition also suppressed the M1-type pro-inflammatory response in AA-induced chronic kidney disease. However, this did not significantly impact upon disease progression arguing that tubulointerstitial damage by M1-type macrophages is not an important pathogenic mechanism in AA-induced chronic kidney disease.

In conclusion, we have demonstrated that AA administration is a potent inducer of JNK signaling in tubular epithelial cells of the kidney. Blockade of JNK signaling with CC-930 significantly reduced the acute effects of high dose AA on tubular cell damage and renal function impairment, presumably *via* inhibition of ROS-dependent cell damage. However, ongoing JNK blockade was unable to protect against DNA damage-induced tubular cell atrophy and senescence that promote chronic kidney disease caused by ongoing exposure to low dose AA.

## Data Availability Statement

The raw data supporting the conclusions of this article will be made available by the authors, without undue reservation.

## Ethics Statement

The animal study was approved (MMCB/2017/21) by the Monash Medical Centre Animal Ethics Committee and performed according to the Australian Code of Practice for the Care and Use of Animals for Scientific Purposes.

## Author Contributions

FY, DN-P, and FM: conceptualization. FY, EO, KL, FM, and GT: methodology. EO and FM: validation. FY, EO, DN-P, FM, and GT: formal analysis. FY, EO, and FM: investigation. FY and DN-P: data curation. FY and XJ: original draft preparation. XJ and DN-P: final manuscript preparation and supervision. FY, XJ, and DN-P: funding acquisition. All authors contributed to the article and approved the submitted version.

### Conflict of Interest

DN-P has previous received funding from Celgene Corporation for studies using JNK inhibitors. No other authors have conflicts of interest.
