# Corrigendum: c-Jun amino terminal kinase signaling promotes aristolochic acid-induced acute kidney injury

**DOI:** 10.3389/fphys.2022.1123475

**Published:** 2023-01-04

**Authors:** Fan Yang, Elyce Ozols, Frank Y. Ma, Khai Gene Leong, Greg H. Tesch, Xiaoyun Jiang, David J. Nikolic-Paterson

**Affiliations:** ^1^ Department of Nephrology, Monash Health and Monash University Centre for Inflammatory Diseases, Monash Medical Centre, Clayton, VIC, Australia; ^2^ Department of Pediatrics, The First Affiliated Hospital of Sun Yat-sen University, Guangzhou, China

**Keywords:** acute kidney injury, chronic kidney disease-, inflammation, c-Jun amino terminal kinase, macrophage, renal fibrosis, senescence

In the published article, there was an error in the **Funding** statement. In the statement, the **Funding** number for the Guangdong Basic and Applied Basic Research Foundation was incorrectly displayed as “019A151501069”.

The correct **Funding** statement appears below.

“This work was funded by the National Health and Medical Research Council of Australia (1058175), the Guangdong Basic and Applied Basic Research Foundation (2019A1515010694), and an award from the China Scholarship Council (FY).”

The authors apologize for this error and state that this does not change the scientific conclusions of the article in any way. The original article has been updated.

